# Recovery of assessed global fish stocks remains uncertain

**DOI:** 10.1073/pnas.2108532118

**Published:** 2021-07-26

**Authors:** Gregory L. Britten, Carlos M. Duarte, Boris Worm

**Affiliations:** ^a^Program in Atmospheres, Oceans, and Climate, Massachusetts Institute of Technology, Cambridge, MA 02139;; ^b^Red Sea Research Centre, King Abdullah University of Science and Technology, Thuwal 23955-6900, Saudi Arabia;; ^c^Computational Bioscience Research Center, King Abdullah University of Science and Technology, Thuwal 23955-6900, Saudi Arabia;; ^d^Department of Biology, Dalhousie University, Halifax, NS B3H 4R2, Canada

**Keywords:** global fisheries, fisheries rebuilding, overfishing, marine resources

## Abstract

Concerns over overexploitation have fueled an ongoing debate on the current state and future prospects of global capture fisheries, associated threats to marine biodiversity, and declining yields available for human consumption. Management reforms have aimed to reduce fishing pressure and recover depleted stocks to biomass and exploitation rates that allow for maximum sustainable yield. Recent analyses suggest that scientifically assessed stocks, contributing over half of global marine fish catch, have, on average, reached or even exceeded these targets, suggesting a fundamental shift in the effectiveness of fisheries governance. However, such conclusions are based on calculations requiring specific choices to average over high interstock variability to derive a global trend. Here we evaluate the robustness of these conclusions by examining the distribution of recovery rates across individual stocks and by applying a diversity of plausible averaging techniques. We show that different methods produce markedly divergent trajectories of global fisheries status, with 4 of 10 methods suggesting that recovery has not yet been achieved, with up to 48% of individual stocks remaining below biomass targets and 40% exploited above sustainable rates. Furthermore, recent rates of recovery are only marginally different from zero, with up to 46% of individual stocks trending downward in biomass and 29% of stocks trending upward in exploitation rate. These results caution against overoptimistic assessments of fisheries writ large and support a precautionary management approach to ensure full rebuilding of depleted fisheries worldwide.

Marine capture fisheries represent the oldest and most extensive human use of the ocean, with a current spatial footprint greater than 4 times that of agriculture (1). Wild-caught fish also provide an important source of protein and micronutrients to over half of the world’s population (2), with an estimated first-sale value of 151 billion US dollars in 2018 (2). With improved technology, however, severe overexploitation has compromised the health of many stocks, causing widespread concern (3). Thus, many countries have made the rebuilding of depleted stocks a cornerstone of contemporary management (3). Likewise, intergovernmental programs like the Convention on Biological Diversity and the United Nations Sustainable Development Goals encourage fish stock recovery to the biomass (*B*) that would sustain maximum sustainable yield (*B*_*MSY*_). This is a common management target that distinguishes stocks below target (*B* < *B*_*MSY*_) from healthy stocks (*B* > *B*_*MSY*_). Despite regional progress (3), however, the United Nations Food and Agricultural Organization (FAO) classifies 34% of the world’s major stocks as below target and unsustainable (2), a proportion that has been increasing ∼4% per decade since the 1970s, with no sign of slowing down.

In contrast to the FAO sample, a subset of stocks with scientific assessments may show more-positive trends. These data-rich populations have formal model-based assessments of biomass and exploitation rate (*U*; the proportion of biomass that is caught every year). They also generally experience better management oversight, and, as such, represent a best-case scenario for the assessment of aggregate fishery status. Recent analyses of a global database of such stocks suggest that, on average, assessed stocks have recovered to near (4) or even beyond (5) internationally agreed management targets since reaching a low point ∼20 y ago. If confirmed, this conclusion would mark a fundamental departure from previous analyses of global fisheries, with significant implications for global fisheries policy and its targets. Here we evaluate the robustness of global recovery by examining a plausible range of alternative metrics for assessing the status of global stocks.

## Results

**Fig. 1.**
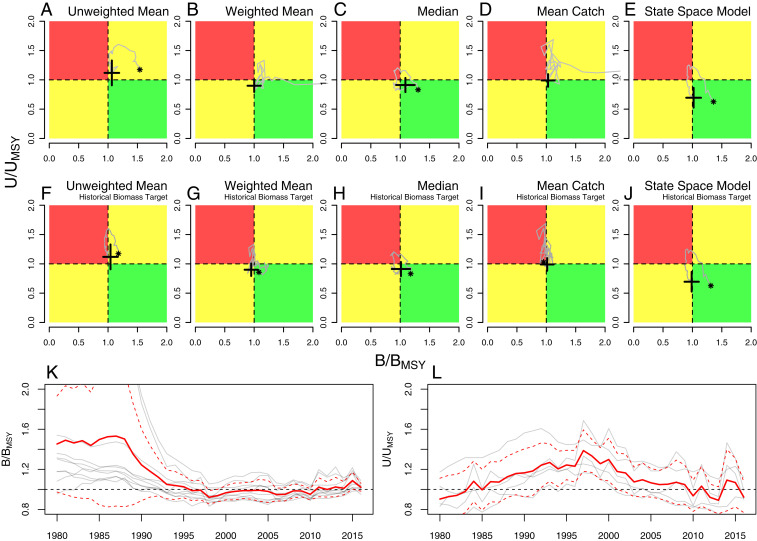
Average global status of assessed fisheries. (*A*–*J*) Each Kobe plot shows the globally averaged biomass relative to the target biomass (*B*/*B*_*MSY*_) on the horizontal axis and the averaged exploitation rate relative to the target exploitation rate (*U*/*U*_*MSY*_) on the vertical axis. The asterisks and crosses show the beginning and end points, in 1980 and 2016, respectively (1980 was out of bounds in *B* and *D*). Cross widths represent SEs. Gray lines give the time series connecting the two end points. The averaging method is labeled on each plot. *K* and *L* give the time series shown in *A*–*J* (gray lines) overlain with the ensemble average and 95% interval (red solid and dashed lines, respectively).

In addition to stocks being classified as healthy (*B* > *B*_*MSY*_) or below target (*B* < *B*_*MSY*_), we also consider whether stocks are currently experiencing sustainable exploitation rates (*U* < *U*_*MSY*_) or overfishing (*U* > *U*_*MSY*_). Stock status relative to these two reference points forms a quadrant, or Kobe plot (Fig. 1), indicating whether stocks are simultaneously healthy and being sustainably fished (green in Fig. 1), whether they are simultaneously below target and experiencing further overfishing (red in Fig. 1), or whether only one of the reference points is satisfied (yellow in Fig. 1). While evaluating the status of individual stocks against these reference points is straightforward, inference about global fisheries status requires statistical methods to aggregate data across many stocks to derive a global trend. This task is complicated by highly variable trends in individual stocks, stocks with missing data in recent years, and large disparities in stock size and total catch.

Of the 10 diagnostics of global stock status considered, we find that 6 support and 4 refute the hypothesis that global stocks have recovered, on average, meaning that both biomass and exploitation rates are now within target levels (i.e., global average *B* > *B*_*MSY*_ and *U* < *U*_*MSY*_; Fig. 1 *A*–*J*). All of the 95% CIs for the 10 metrics and their ensemble average straddle the *B* = *B*_*MSY*_ line, while 8 metrics and the ensemble average straddle the *U* = *U*_*MSY*_ line, indicating low statistical confidence in recovery conclusions from any single metric. The ensemble average across the 10 methods shows that stock status is only marginally above target, with oscillations above and below the target in recent years (Fig. 1 *K* and *L*). These results contrast with the high confidence in recovery reported recently (5). Using the precautionary biomass target of half the historical biomass (4, 6), we see a consistently negative impact on assessments of global stock status (Fig. 1 *E*–*H*). *B*_*MSY*_ targets estimated within the stock assessments are lower, on average, than half the historical maximum biomass, which allows for greater stock depletion to be considered sustainable (6). However, stock assessment *B*_*MSY*_ is estimated with considerable error from historical time series (7) that may no longer represent current ecosystems (8–11). Repeating these analyses for time series of spawning stock biomass yielded similar conclusions (*SI Appendix*).

**Fig. 2.**
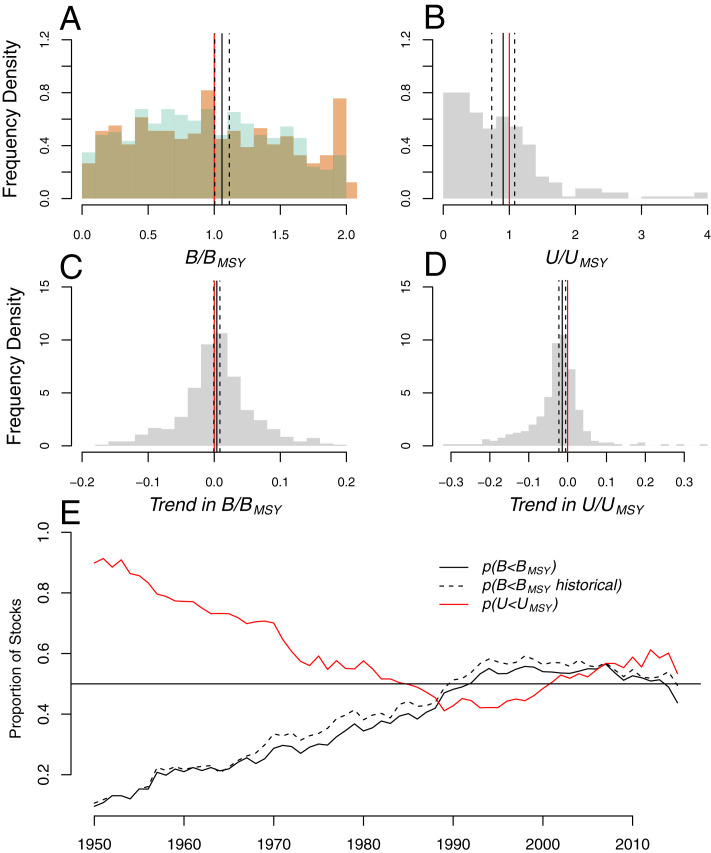
Status and trends across individual fish stocks. *A* and *B* give the distribution of the most recent estimate of (*A*) *B*/*B*_*MSY*_ and (*B*) *U*/*U*_*MSY*_ across individual fish stocks. Green and orange overlapping histograms in *A* give *B*/*B*_*MSY*_ using the stock assessment estimated *B*_*MSY*_ and half the maximum historical biomass, respectively. *C* and *D* give the distribution of trends in (*C*) *B*/*B*_*MSY*_ and (*D*) *U*/*U*_*MSY*_ for individual fish stocks over the most recent 10 y of assessment data (units of per year). Solid and dashed black lines in *A*–*D* give the means and 95% intervals across averaging methods from Fig. 1. *E* gives the time series for the proportion of stocks within biomass (black) and exploitation rate (red) targets since 1950.

Considering recovery trends at the individual stock level, we find that up to 48% of stocks remain below biomass targets and 40% of stocks remain above exploitation limits (Fig. 2 *A* and *B*) as of 2016, representing the last year with sufficient data. Examining trends in *B/B*_*MSY*_ and *U/U*_*MSY*_ for individual stocks over the most recent 10 y of available data, we find that up to 46% of stocks are trending downward with respect to biomass targets and 29% are trending upward in exploitation rate (Fig. 2 *C* and *D*). Notably, average biomass trends across stocks are only marginally different from zero. The maximum historical proportion of stocks below biomass targets was 57% in 2007, and the maximum proportion of stocks over exploitation targets was 59% in 1989, indicating only modest improvements of 9% and 19%, respectively (Fig. 2*E*).

## Discussion

Our results demonstrate that inference about the status of global fish stocks depends on specific choices in deriving trends across highly variable stocks. Considering trends in individual stocks and an ensemble of statistical summaries, we find a higher degree of uncertainty than has been reported previously (5). Choosing the “right” averaging procedure remains ambiguous and will depend on personal or policy preferences—for example, whether stocks should be weighted according to stock size (emphasizing food supply) or weighted equally (emphasizing species diversity); for example, see ref. 12 vs. ref. 13.

Understanding the dynamics of individual stocks involves several uncertain quantities, including biomass (14), reference points (6, 7), and productivity (11), all complicated by climate change and other factors that alter these quantities over time (8–10). To accommodate this uncertainty, we suggest that inferences about global stock status be made using an ensemble of diagnostics, as attempted here. Such an approach is used in other fields, including climate studies (9), where comparing different models provides a means to quantify complex uncertainties. Alternatively, the more conservative scenarios within the ensemble may be adapted to conform to a precautionary approach, which requires managers to err on the side of caution when multiple interpretations of the data are plausible. Unfortunately, history has shown that there are strong short-term incentives to follow the most optimistic scenario that allows for greater exploitation rates, a systematic bias that has contributed to past stock collapses (15). Similar ensemble approaches can be applied at the regional level (5) where trends in stock status may deviate from the global average due to differences in species composition, data quality, and differences in the intensity of historical overfishing.

In conclusion, we caution against overoptimistic assessments of global fisheries based on individual diagnostics that do not capture the complexity and uncertainty of global fisheries datasets. While exploitation rates appear to be declining in most assessed stocks, observed trends in biomass are variable and uncertain; thus continued efforts to constrain exploitation rates below *U*_*MSY*_ are required to ensure that fish biomass unambiguously recovers beyond appropriate contemporary targets.

## Materials and Methods

We characterize the distribution of recovery rates across individual stocks and consider an ensemble of 10 aggregate statistical metrics for $B/B_{MSY}$ and $U/U_{MSY}$ used in previous literature. The 10 metrics include 5 statistical averaging methods and 2 rebuilding targets. Statistical averaging methods included biomass-weighted and unweighted averages, catch-weighted averages, medians, and a hierarchical state space model smoother. Rebuilding targets included stock assessment estimated reference points, where available, and half the historically estimated maximum biomass. We also characterize the proportion of stocks within target limits over time. See *SI Appendix* for further details.

## Supplementary Material

Supplementary File

## Data Availability

Fisheries stock assessments are publicly available in the Ransom Aldrich Myers (RAM) Legacy Stock Assessment Database in Zenodo (https://doi.org/10.5281/zenodo.3676088).
