# Supplementary material for: Recovery of assessed global fish stocks remains uncertain
Source: Proc Natl Acad Sci U S A. 2021 Jul 26;118(31):e2108532118. doi: 10.1073/pnas.2108532118 (PMC8346865; doi:10.1073/pnas.2108532118)
Supplement: Supplementary File [file pnas.2108532118.sapp.pdf]

PNAS
www.pnas.org

Supplementary Information for
Recovery of assessed global fish stocks remains uncertain

Gregory L. Britten[1*], Carlos M. Duarte[2], Boris Worm[3]

[1]Program in Atmospheres, Oceans, and Climate, Massachusetts Institute of Technology, Cambridge, USA
[2]King Abdullah University of Science and Technology, Red Sea Research Centre and Computational Bioscience Research Center, Thuwal, Saudi Arabia
[3]Department of Biology, Dalhousie University, Halifax, Canada

[*]**Email:** gregleebritten@gmail.com

This PDF file includes:

Supplementary Information Text
SI References

Supplementary Information Text

Methods

We analyzed the RAM Legacy Stock Assessment Database version 4.491 (https://zenodo.org/record/3676088). Where available, we extracted time series of exploitation rate (U) and total stock biomass (B). We also extracted stock assessment estimates of the biomass and exploitation that are estimated to produce maximum sustainable yield, B_{MSY} and U_{MSY}, respectively, where available. We calculated multiple statistical aggregations of the time series to examine trends in average biomass and exploitation rate over time. Five aggregate statistics were used: biomass-weighted, biomass-unweighted averages, catch-weighted averages, and medians for B/B_{MSY} and U/U_{MSY} ratios. Biomass and catch weights were taken as the mean historical biomass and catch, respectively, for each stock over the history of the available time series. We also applied the state space model smoother developed in (1). This method assumes individual time series follow a common trend and extrapolates missing data for individual stocks. We refer to the original paper for the mathematical description (1). For targets, we used the stock-assessment estimated reference points where available, or alternatively half the historically estimated maximum biomass. The combination of five aggregate statistics and two alternative targets gives ten total recovery metrics. The alternative target was motivated by the well-known Gordon-Schaefer fisheries production model, which predicts B_{MSY} as half the stock carrying capacity (2), which we approximated by the maximum historically observed annual biomass. This alternative target is attractive as a simple metric that is available for all stocks and avoids many uncertain mathematical assumptions about contemporary stock-specific population dynamics. We calculated the weighted standard deviation for the biomass-weighted and catch-weighted means, the simple standard deviation for the unweighted mean, and the median absolute deviation for the median. Standard errors for the aggregations were calculated by the dividing each measure of variation by the square-root of the number of stocks with data available each year. We repeated all analyses using estimates of spawning stock biomass and reached similar conclusions (see https://github.com/gregbritten/fisheries_uncertainty_public). Code developed for this paper is available at https://github.com/gregbritten/fisheries_uncertainty_public.

SI References

1. R. Hilborn, *et al.*, Effective fisheries management instrumental in improving fish stock status. *Proc. Natl. Acad. Sci. U. S. A.* **117**, 2218–2224 (2020).
2. D. Pauly, R. Froese, MSY needs no epitaph — but it was abused. *ICES J. Mar. Sci.* **fsaa224**, 1–7 (2020).
